# The Current Number

**Published:** 1884-08

**Authors:** 


### THE CURRENT NUMBER.

This number of the INDEPENDENT PRACTITIONER contains seventy two pages. The last number was one of sixty-four pages, and yet we cannot meet all the demands upon our space. We have laid aside a part of the editorial matter that had been prepared, and have cut down the space devoted to "current news" that we might give to correspondents the room that they demanded. Our only aim is to make a journal that shall properly represent the profession, and that shall be entirely devoted to those whom it represents. The class of articles that we accept and publish is therefore a broad one, for there are many interests to serve. Independent as we are of all manufacturing or mercantile interests, and of all schools and other organizations, we have felt it a duty that we owed to the profession to present many things that might otherwise have been unsaid. When an article has been received we have not enquired whether it was in accord with the belief or prejudices of the majority of dentists, nor even if it were palatable to individuals, no matter whether they were or were not personal friends and supporters of the journal. Our sole enquiry has been if the matter were likely to result in good to the whole profession. We think no one has hesitated to send communications that were opposed to the known and expressed convictions of the publishers, for we have not attempted to make this a personal organ, and have no pet theories that we wish to advance. This is a journal that belongs to the profession, and all shades of opinion shall have opportunity for hearing. Whatever will, in our judgment, advance the interests of dentistry, shall have place. We therefore welcome articles upon any subject connected with dentistry, but as our space is limited, they may sometimes have to wait their turn for publication. That so many have been sent us is an indication that the INDEPENDENT PRACTITIONER is meeting with a large degree of popular favor, and is filling a needed place in our professional literature.

